# Environmental and Human Microbiome for Health

**DOI:** 10.3390/life12030456

**Published:** 2022-03-19

**Authors:** Bimala Panthee, Saroj Gyawali, Pratiksha Panthee, Kuaanan Techato

**Affiliations:** 1 Faculty of Environmental Management, Prince of Songkla University, Songkhla 90112, Thailand; bsgyawali@gmail.com; 2 Sustainable Study and Research Institute, Kathmandu 44600, Nepal; pantheepratiksha@gmail.com

**Keywords:** environmental microbiome, human microbiome, health effects, pathogen, commensal, diversity, nature

## Abstract

Microorganisms are an essential part of life on the earth and can exist in association with virtually any living thing. The environmental microbiome is much more diverse than the human microbiome. It is reported that most microbes existing in the environment are difficult to culture in the laboratory. Whereas both pathogenic and beneficial microbes may be prevailing in the environment, the human body can have three categories of microbes- beneficial, pathogenic, and opportunistic pathogenic. With at least 10-fold more cells than human cells, microbes as normal flora are critical for human survival. The microbes present in the human body play a crucial role in maintaining human health, and the environmental microbiome influences the human microbiome makeup. The interaction between the environmental and human microbiome highly influences human health, however it is poorly understood. In addition, as an established infection is associated with health-seeking behavior, a large number of studies have focused on the transmission and dynamics of infectious microorganisms than the noninfectious or beneficial ones. This review will summarize how the interaction between the environmental and human microbiome affects human health and identify approaches that might be beneficial for humans to improve health by being exposed to the natural environment.

## 1. Introduction

Microorganisms, the oldest living organisms in the biosphere, are omnipresent, critical to the surroundings, and linked with good and ill health effects. In nature, microorganisms have an essential role in biochemical cycles, such as nitrogen, phosphorous, and carbon. Microorganisms are vital for nitrogen fixation, assimilation, mineralization, nitrification, and denitrification. Similarly, they participate in the phosphorus cycle by mineralization, assimilation, precipitation of phosphorus compounds [1] and in the carbon cycle by converting atmospheric carbon dioxide into organic material [2]. They further play a vital role in human survival by contributing more enzymes or proteins responsible for human survival than humans themselves do. It is estimated that the human body harbors more than 10 trillion living microorganisms [3], at least ten times more than the number of human cells itself [4]; the precise role of each is difficult to understand. The microbes associated with the human body are the major contributor to host metabolism by providing essential micronutrients, such as vitamins and other metabolites. For example, gut microbes produce essential micronutrients, vitamin K and enzymes, allowing humans to digest foods and absorb various essential nutrients [4]. Microbial diversity in the environment is much higher than the diversity inside humans, suggesting that a variety of new microbes are found in the environment. Despite extensive studies, a vast majority of microbes are under-discovered, and so is their effect on human health.

Given that humans are constantly exposed to various microorganisms in the environment, which comprises beneficial and pathogenic microbes, it is crucial to understand their physiological role. The health-seeking behavior of human beings has dramatically facilitated the identification of various novel pathogenic microbes. It is evident that the disease-causing microbes have an apparent effect, obtain immediate attention, and are identified earlier than the beneficial ones. Such microbes cause illnesses that need to be cured before the infections prove to be fatal. Therefore, their identification and detailed studies to understand their nature, pathogenicity, virulence factors, and susceptibilities to existing antimicrobial agents are studied as soon as they appear and start causing problems. On the other hand, beneficial microorganisms that, in the long run, help solve issues associated with lifestyle diseases and mental well-being do not come into immediate attention.

Additionally associated with the human body are opportunistic pathogens which reside as commensals and do not cause diseases under normal circumstances. These are actively looking for opportunities to infect the host and, upon sensing conditions, such as decreased body immunity. The beneficial microbes protect against colonization of opportunistic pathogens and serve as an essential barrier to reduce human exposure to an infectious or otherwise harmful agent. Any dysbiosis in these dynamics is expected to affect the human health. In addition, beneficial microbes in the environment could act as a modulator of the microbiome inside the human body. However, based on the recent trend of increasing migration towards the developed regions, the United Nations (UN) estimates that nearly two-thirds of the world population will live in cities by 2050. Although this transition has several sound effects, it is expected to change the land use pattern and policies, transform agricultural land to build megastructures, and increase the loss or the fragmentation of green spaces in the designated urban areas, directly impacting environmental microorganisms. Exploring the relationship between the environmental and human microbiome could improve our understanding of both beneficial and disease-causing microbes.

This review will first explore the microorganisms found in the environment and inside the human body. Next, we will evaluate and discuss how these microbes can affect human health, including infections and beneficial effects. Finally, after summarizing the current evidence, this review will suggest the gaps that need to be filled.

## 2. Diversity of Microbes in the Environment and Human Body

Conservation, stability, and maintenance of global genetic resources and ecosystems require maintaining microbial diversity [5,6]. An analysis performed during early 2000 estimated that more than 50 bacterial phyla exist in the environment [7]. Interestingly, about half of these have not been cultured in the laboratory, indicating that microbial growth in the natural and indoor environments or laboratory is different. Microbial diversity is higher in the outdoor environment as it represents diverse species associated with animals, plants, livestock, and other factors, such as soil and air [6]. Although the number of microbial cells present in the human gut and soil is similar per gram, soil contains more diverse species than the human gut. For instance, 4 × 10^3^–5 × 10^4^ species are found in one gram of soil, and 4 × 10^2^ species are found in one-gram feces of humans [8].

Furthermore, the soil depth also determines the density of the bacterial community, with the highest densities found above 30 cm and the lowest below 60 cm [9]. Forest soil contains higher bacterial diversity (2–5 times) than agricultural organic soil. Agricultural organic soil has higher diversity than agricultural sandy soil [10], suggesting that environmental stress and agricultural management determine the richness of microbial diversity. In addition, soil bacterial abundance varies according to carbon input, temperature, soil depth, and hydration status [11]. The abundance of microorganisms varies depending upon whether they belong to agricultural and forest soil, wetlands, grass, and desert soils [12]. Apart from that, sewage as an indicator of the human microbiome can be used broadly to obtain an idea of the microbiome of humans residing in a particular area [13,14,15]. In addition, the diversity of human-associated microbial community would let one know about the presence of pathogenic microbes that cause immediate infectious diseases or are associated with chronic condition, which allow us to take timely actions [16,17].

On the other hand, the indoor environment is mainly associated with human activities and non-living materials that can promote or retard microbial growth. The sources of microbes in the built environment are limited to soil, skin, pets, outside air, vagina, and gut, hence representing a lower diversity [18,19]. Likewise, microbial richness varies between body sites, individuals, and age within the human body. The well-known body sites for microbial colonization in the human body are the gut, skin, oral cavity, respiratory tract, and vagina [20].

## 3. Beneficial Microbes Present in the Human Body

As discussed, microbes can be both beneficial and pathogenic to humans. Microbes can be helpful in different ways, for instance, by preventing pathogen colonization, modulating the immune system, digesting nutrients, detoxifying, and producing nutrients, stimulating cellular differentiation, improving barrier function, and altering the gut–brain axis [21]. Similarly, a healthy host-microbiota relationship confers normal regulation of cardiovascular and digestive systems, resistance to pathogen colonization and supports host for defense, and anti-inflammatory, metabolic, and antioxidant potential become available [22]. Thus, microbes found in different organs of the body act in various ways to benefit the host. Table 1 shows the abundance of microorganisms based on the site in the human body and their effects on human health. It was found that a higher number of beneficial microbes are located in the gut, followed by the respiratory tract (Table 1). Actinobacteria, Bacteroidetes, Firmicutes, or Proteobacteria were the commonly found microorganisms throughout the human body [23,24,25].

Gut microbiome: The gut accounts for a large number of microbes that are required for the processing of ingested food. Gut bacteria, such as *Lactobacillus*, *Enterococcus*, and *Bifidobacterium* are essential for maintaining epithelial integrity, enhancing the intestinal barrier, protecting chemical-induced disruption of the epithelial barrier [26,27], and for normal development and functioning of the immune system and central nervous system [26]. Some microbes colonize immediately after birth [28,29], and many are obtained from the mother via breast milk. Therefore, the function and composition of the microbiome in an infant are greatly determined by the life events, and, more interestingly, the infant microbiome becomes comparable to the adult microbiome by the age of 2.5 years [30], suggesting that the early age is crucial for maintaining the microbial diversity. The gut microbiome is the largely studied field where relationships of the gut microbiome with human behavior and mental health have been established.

Oral microbiome: The oral cavity harbors the second most diverse microbial community (above 700 species) after the gut. However, most of them have not been cultured [22,24,31]. In analyzing the healthy oral cavity, six different bacterial phyla, namely *Firmicutes*, *Actinobacteria*, *Proteobacteria*, *Bacteroidetes*, *Fusobacteria*, and *Saccharibacteria* were identified with higher diversity in tonsils followed by tooth surface, and the least diverse microbes were found in the maxillary vestibule [24]. Common microbes in the oral cavity are Streptococcaceae, Veillonellaceae *Streptococcus mutants*, *Porphyromonas gingivalis*, *Staphylococcus*, and *Lactobacillus* [25,32]. *S. mutants* and *P. gingivalis* are pathogenic bacteria mainly responsible for dental plaque and caries, while *Lactobacillus* is the beneficial bacteria that can ferment sugar to produce lactic acid [32]. Interestingly, species associated with periodontal diseases, such as dental caries and deep dentin were not detected in healthy teeth and oral cavities [24]. This suggests that the microbial composition of the oral cavity affects oral health.

The microbiome of the respiratory tract: Normal healthy adults breathe more than 7000-L of air every day [33]. It is expected that around 2000 different microbes exist in the air. This indicates that an enormous number of microbes present in the air enter the respiratory tract as we breathe. The analysis of respiratory microbiota using genomic techniques reveals that Actinobacteria, Firmicutes, and Proteobacteria are the most common phyla found in the nasal cavity [25]. Overall, the oropharynx and nasopharynx contain diverse bacterial communities comprised of streptococcal species, such as *Neisseria* spp. *Rothia* spp., and anaerobes, including *Veillonella* spp., *Prevotella* spp., and *Leptotrichia* spp. [34]. The availability of bacteria in the upper respiratory tract changes according to age, host immune response, olfactory function, and smoking habits [33]. It is important to note that the upper respiratory tract works as a gatekeeper for respiratory health. The microbial composition in the upper respiratory tract resembles the lung microbiota in healthy individuals [34].

Vaginal microbiome: Lactobacilli are common bacteria found in a healthy vagina where *Lactobacillus* sp. safeguard the vaginal environment from non-indigenous and potentially pathogenic microorganisms [35]. The richness and diversity of bacteria changes according to the pregnancy status as evidenced by reduced diversity with the dominance of *Lactobacillus* followed by *Clostridiales*, *Bacteoidales*, *and Actinomycetales* [36].

Skin microbiome: Staphylococcus and Micrococcus are the most prevalent isolates in the skin. The members of the skin microbiota are characterized by their ability to metabolize amino acids, steroids, lipids, and sugars [37]. The diversity of bacteria in the skin depends upon its moisture level—moist, sebaceous, and dry areas of skin harbor different microbes. Where the least diverse microbes are found in sebaceous sites, e.g., forehead, retro auricular crease, alar crease, and the back, most diverse microbes are found in dry areas, e.g., volar forearm, different locations of the hand and the buttocks [23]. The higher diversified bacteria available in the dry skin sites might be associated with frequent exposure of these sites to the external environment [38]. However, it is still unknown how skin microbes can survive or replicate on the skin and are frequently encountered in the environment [23].

## 4. Factors Associated with Microbial Dysbiosis and Its Impact on Human Health

**Figure 1:**
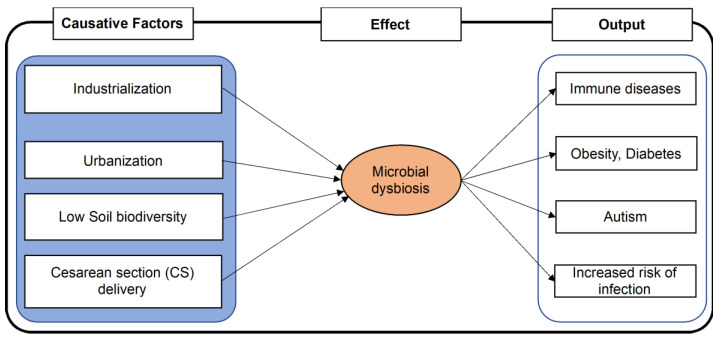
Factors associated with microbial dysbiosis leading to disease. A change in lifestyle and food habits associated with industrialization and urbanization, and cesarean delivery is expected to reduce humans’ microbial balance and diversity, leading to the appearance of several non-communicable diseases and ill effects in health.

The diversity of beneficial microorganisms in the human body has a crucial role in maintaining a healthy status. Conversely, lower diversity of such microbes or higher diversity of pathogenic microbes in the body is a sign of ill-health (Figure 1). A study found that women who had bacterial vaginosis had complicated vaginal infections with microbial dysbiosis and the presence of several newly recognized potential pathogenic bacterial species [64]. Similarly, changes in gut microbe composition are thought to be responsible for various diseases, including autoimmune disease, diabetes, inflammatory bowel disease, psoriatic arthritis, eczema, coeliac disease, and arterial stiffness [65,66]. An intervention study identified that the gut and skin microbial diversity greatly varied by children’s interaction with nature, such as soil and plants [67]. In addition, the diversity of microbial communities close to nature was found to be associated with an increase in immunoregulatory pathways [67].

Similarly, mimicking farm-like increased microbial diversity in non-farmhouses led to a reduced risk of asthma [68]. In addition, a separate study found that the gut microbiome diversity and maturation in infants provided a protective effect against asthma [69].

Moreover, the role of nutrition in maintaining the balance of the microbiome seems crucial as nutritional changes in a lifetime may lead to microbial dysbiosis and increased incidence of chronic inflammatory disease and obesity [66]. Furthermore, people with microbial dysbiosis are more sensitive to environmental changes, while those with a balanced microbiome can maintain their health even in adverse environmental conditions [70]. In the same line, individuals living in a complex, species-rich ecosystem can have more diversified and balanced microbiomes and be more resistant to the disease [71]. Thus, it is essential to have a balanced and diversified microbiota in the body.

## 5. Factors Associated with Microbial Diversity in the Human

Multiple factors might affect the microbial balance inside a human being. Therefore, this review will mainly focus on the six different factors possibly involved in changing the abundance, diversity, and balance of microorganisms inside or on the surface of the human body.

Living with pets: Living with pets differently affects the microbiome. For example, Kates et al. [72] identified that adults living with pets tend to have a microbiome with beneficial behavior. In contrast, Azad et al. [73] found that microbiota richness and diversity tended to be increased in infants living with pets but tended to have a higher number of pathogenic microbiomes than beneficial. However, prenatal pet exposure significantly increased microbiomes that show beneficial behavior and significantly decreased pathogenic microbiome, suggesting that prenatal pet exposure can benefit for the newborn [74].

Living with the environment: The biodiversity hypothesis explains that the frequent contact of people with the natural environment can increase the diversity in the human microbiome, promote the immune balance and protect the individual from allergy and inflammation [75]. For instance, people living in urban and rural have different degrees of exposure to microorganisms from the soil, nature, water, and biomasses used in agriculture or livestock, which is associated with a difference in their skin [38] and gut microbiome [76]. In line with this, Hanski and collaborators [77] established the relation between exposure to the environment and skin atopy. Furthermore, atopy was significantly associated with environmental biodiversity around the house, with decreased incidences among people who had flowering plants in the yards and lived nearby forest and agricultural land. Furthermore, it has been identified that children who grow up on farms in contact with livestock or those who have exposure to dogs or certain microbes early in life have reduced incidences of allergic diseases and asthma in later life [78,79,80,81,82]. In addition, the microbiota of individuals in long-term care facilities was much less varied than those in the community dwellers [83].

Similarly, urban green space is also positively associated with biodiversity, followed by a healthy environmental microbiome associated with a healthy human microbiome leading to immunological resilience and consequently good health and well-being. Urban green space also has other ways for good health and well-being through thermal buffering, air cleaning, social integration, calming environments, physical activity, and food gardens [84]. In summary, all these studies highlight the importance of the natural environment for the well-being of humans. World Health Organization also emphasized that “reduced contact of people with the natural environment and biodiversity, and biodiversity loss in the wider environment, leads to reduced diversity in the human microbiota, which itself can lead to immune dysfunction and disease”.

Industrialization: With rapidly progressing industrialization, more people live in industrialized urban areas of the world. These people are expected to live in crowds and have less contact with nature. This further leads to low microbial diversity related to their eating behaviors, disruption of the biological clock, use of antibiotics, the higher practice of cesarean section (CS) delivery during childbirth [85]. This, eventually, is associated with the higher prevalence of immune diseases, metabolic diseases, colorectal cancer, and autism [85]. Furthermore, urban life is also characterized by a sedentary lifestyle and associated with reduced contact with nature, thereby changing the body’s microbial community [75].

Method of delivery: During the first three days of life, infants’ microbial colonization is substantially affected by the mode of delivery. This was evidenced by the absence of Bifidobacteria sp. among infants born by cesarean section and the presence of subject-specific microbial profile among infants born by vaginal delivery though predominant groups were B. longun and B. catenulatum [86]. Therefore, it is expected that during normal vaginal delivery, the newborn infants come in contact with maternal vaginal microbiota, which will later grow and mature in the child.

Soil biodiversity: Soil biodiversity benefits human health by providing clean water, food, and air by suppressing the disease-causing soil organism [87]. Even though environmentally healthy soil and the human gut have a roughly similar number of active microorganisms, the diversity of the human gut microbiome is mere 10% of that of soil biodiversity [8]. This indicates that human microbial diversity can further be enhanced by interacting with natural healthy soil. However, the current modern lifestyle, including agrochemical, low plant biodiversity, inappropriate soil management practices in rural areas, has decreased soil microbial diversity [8].

Age: Age affects microbial diversity. In most cases, age is positively correlated with diversity. By the age of 3, the gut microbiome’s composition starts to resemble that of adults [30]. Whereas age is positively correlated with the higher microbial diversity in normal-weight children, this was negative among obese and overweight children suggesting that child weight may impair the microbial diversity [88]. Interestingly, one study found higher diversity among young adults, but the same was not found among middle-aged adults [89]. In summary, it suggests that the health condition of young adults and middle-aged adults should be considered differently.

Food consumption: Foods consumed in the form of plants, vegetables, fruits, seeds also determine human microbiota. Plants have their own microbial community in the form of either endophytic bacteria or rhizobacteria. Both kinds of plant microbiome are beneficial to plants to improve plant growth, promote resistance towards biotic and abiotic stresses, and produce metabolites with medicinal properties [90,91]. It has been found that high fruit and vegetable intake was positively associated with the abundance of *Faecalibacterium prausnitzii*, *Akkermansia muciniphila*, *Ruminococcaceae*, Clostridiales, Acidaminococcus, and Bifidobacteria [92,93], while negatively associated with Firmicutes [94] highlighting that diet and specific dietary components could affect microbiota composition, diversity, and activity. In addition, consumption of fermented foods is another direct source of the microbial community that changes human microbiota significantly.

Thus, the interrelationship between the environmental and human microbiome is complicated. Maintaining biodiversity seems crucial for the balanced microbial ecosystem within the human body and the environment. With the evidence of a positive association between microbiome-rich environmental surroundings and the good health of people, the focus should be paid to creating the natural environment as much as possible to prevent allergic and chronic non-communicable diseases.

## 6. Environment-Host Dynamics

The disease can occur according to the condition of the host environment, and the relationship between host, pathogen, microbiome, and the environment determines the disease outcome [71]. In normal conditions, the human microbiome stays in its respective place and helps the organism adapt to its surroundings, protects it from diseases, and helps in physiological functioning. Similarly, by preventing microbial dysbiosis of the ecosystem and contributing to ecological activities, the environmental microbiome promotes the ecosystem’s stability and biodiversity. Thus, microbiomes of the host and the environment are interlinked and exchange bacteria on a regular basis [45]; for example, humans obtain microbes via means of food, or their interaction with the environment and environment receives microbes from humans in the form of human excreta. The entry of environmental pathogenic microbiomes inside the human body allows the host-microbiome to combat the pathogenic microbiome. The human microbiome changes during the immune-compromised state, changed diet, antibiotic treatment, stress level, and changes in the external environment. The best example for the host status of the host environment can be explained by *C. difficile*, which is a well-studied microorganism responsible for colitis. In normal conditions, they are deficient in number in the gut because gut microbiota provides colonization resistance against *C. difficile*. Conditions, such as antibiotic use, diminish the number of beneficial microbiota, eventually increasing *C. difficile* growth leading to disease [95].

## 7. Improving Health: Living with Environment

With changes in human lifestyle and declining microbiome, it is crucial to focus on maintaining the microbiome health of the human being. The decrease in biodiversity and declination of the ecological balance has led to the Emerging Zoonotic Diseases (EZDs), which threats human, animal, and environmental health [96]. The health of humans is interrelated with the health of animals which, in turn, depends upon the food consumed and the environment shared. This comprises of incorporating One Health approach, which takes into account both pathogenic and non-pathogenic microbial transmission between humans, animals, and the environment [97] with the fact that environmental microbiome, as well as the microbiome of animals in close contact, can affect both the human microbiome and human health. For instance, a significant positive correlation between salmonella abundance in the municipal waste sample and the number of salmonellosis disease prevalence in the community [16] suggests that environmental health can predict human health. Similarly, the early life exposure of humans with pets can be a protective factor for the health in later life. However, it also depends on the health of the pets, which may affect the health of humans. Likewise, encroachment of wildlife by humans has opened up another aspect where humans are in closer contact with wild animals than before, increasing the likelihood of interaction with diverse microbial communities.

**Figure 2:**
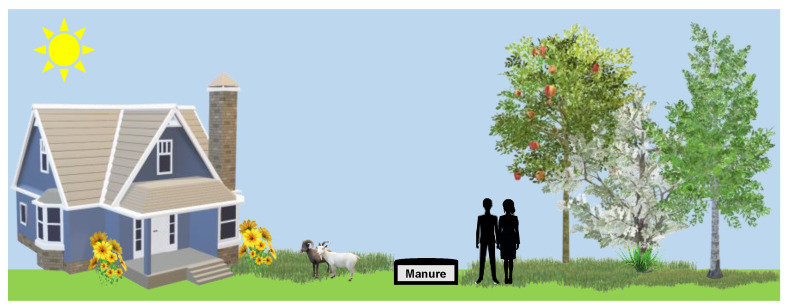
A schematic representation of living with the environment for a diversified and balanced microbiome. A close living with the natural environment with domestic or pet animals, diverse soil, flowering plants in the yard, and close proximity of forest can facilitate the diversification and balance of human microbiota.

As our understanding of microbial community in the environment increases, we have become more aware of the benefits that environmental microbes can provide to our health. Evidenced by several studies is the influence of environmental microbes upon the human microbiome and ultimately human health. As the living environment dramatically affects the microbiota, a closer living with nature would facilitate the diversification and balance of microbiota inside the body (Figure 2). A multi-disciplinary understanding, joint effort, and thought system can be the possible solution to obtain optimum health for humans, animals, and the environment.

## 8. Summary and Conclusions

The interaction between the human microbiome and environmental microbiome will shape the human microbiome diversity and composition, which in turn affects the overall human health, both physical and mental. As science in advancing toward next-generation sequencing technologies, identification and study of a large number of microorganisms in a short time is achievable. Consequently, microorganisms that are not easily cultured in laboratory-derived artificial mediums are being identified. With the identification of a large number of microorganisms, the studies for the understanding of their role in nature and human health have become important. In addition, with the changing environmental conditions and urbanization, the composition and diversity of the environmental microbiome are also changing.

Moreover, the meaning of domestic animals has been changing and confined to pet animals rather than farm animals, which used to be the case before urbanization. This has led to changes in the diversity of interaction of animals and humans. Animals have their own microbiome, and as the types of animals that interact with humans within the modern era has changed, so did the diversity and composition of the microbiome that humans are exposed to. Similarly, the dietary pattern is also equally important to have the beneficial microbial diversity evidenced by the higher diversity found among people who eat more vegetables and fruits. Hence, interacting more with farm animals, increasing the consumption of plant-type food (vegetables), including fruits, and creating a natural or farm-like environment in the homes to improve the interaction with the environmental microbiome is essential. The diversity and composition of farm animals and plants are also impacted due to changes in their diet, environment, and methods of rearing and/or breeding.

To maintain the balance between environmental and human microbiome, a multi-sectorial approach is needed, considering the inherent role of microorganisms in their natural niche. Attempts should be made to preserve the beneficial organisms present in the environment and within the host by investigating the dynamics of the relationship between the environmental microbiome and humans. In addition, industrialization with proper environmental management and maintenance of environmental surroundings as close to natural as possible and improving lifestyle pattern is the emergent need in the current global scenario.

## Figures and Tables

**Table 1:** Microorganisms found in different parts of the body and their possible effect on human health. Genera tending to fall towards pathogenic and beneficial are indicated by bold and italic, respectively. The * sign next to the genera names indicates that these beneficial microbes are also reported to cause infection; genera that are neither bold, italic, nor have * symbol are unclassified (The list regarding beneficial and pathogenic effects is not exhaustive).

Body Sites	Common Phyla	Common Genera	Positive Effects of Beneficial Genera
Gut [39]	Actinobacteria	Corynebacterium *	
		*Bifidobacterium*	Stimulates immune system, Gut homeostasis, Protection against gastrointestinal infection [40,41,42,43,44], Protective role in TNF-α induced inflammatory response [45].
		**Atopobium**	
	Firmicutes	*Faecalibacterium*	Prevention of Inflammatory bowel disease and colorectal cancer, Protection of colon, control of metabolism [46], Immune response/balancing immunity in intestine [46,47].
		Clostridium *	
		*Roseburia*	Immunity maintenance, Anti-inflammatory response [48,49,50].
		**Ruminococcus**	
		**Dialister**	
		*Lactobacillus*	Anti-microbial activity [51,52], Cholesterol metabolism, immunomodulation, anti-allergic effects, anti-diabetic effects [51].
		Enterococcus *	
		**Staphylococcus**	
	Bacteroidetes	**Sphingobacterium**	
		Bacteroides *	
		**Tannerella**	
		Parabacteroides	
		Alistipes	
		**Prevotella**	
	Proteobacteria	**Escherichia**	
		**Shigella**	
		**Desulfovibrio**	
		**Bilophila**	
		**Helicobacter**	
	Fusobacteria	**Fusobacterium**	
	Verrucomicrobia	Akkermansia *	
Oral cavity [53]	Actinobacteria	**Actinomyces**	
		**Atopobium**	
		Corynebacterium *	
		**Rothia**	
	Proteobacteria	**Campylobacter**	
		**Haemophilus**	
		**Neisseria**	
	Bacteroidetes	**Bergeyella**	
		**Capnocytophaga**	
		**Prevotella**	
	Firmicutes	**Granulicatella**	
		**Streptococcus**	
		*Veillonella*	Lactate metabolism, NO_2_ production, Maintain oral health and general health [54]
	Saccharibacteria		
	Fusobacteria	**Fusobacterium**	
Respiratory tract [25,33]	Actinobacteria	Corynebacterium *	
		**Cutibacterium**	
		*Bifidobacterium*	Reduction in respiratory tract infections [55,56,57] Reduces the colonization of pathogenic bacteria [55]
		**Rothia**	
	Firmicutes	**Dolosigranulum**	
		**Staphylococcus**	
		Veillonella *	
		Lachnospiraceae	
		**Streptococcus**	
	Bacteriodetes	**Prevotella**	
	Fusobacteria		
	Proteobacteria		
Vagina [58]	Actinobacteria	**Gardnerella**	
		**Atopobium**	
		Eggerthella	
	Firmicutes	Alloiococcus	
		Papillibacter	
		*Megasphaera*	
		**Aerococcus**	
		*Lactobacillus*	Immunomodulation and restoration of healthy microflora in the vagina, The first line of defense against vaginal pathogens [59,60].
		**Streptococcus**	
	Bacteroidetes	**Prevotella**	
	Fusobacteria		
Skin [61]	Actinobacteria	**Propionibacterium**	
		**Corynebacterium**	
		**Micrococcus**	
		**Mycobacterium**	
		**Kocuria**	
		**Rothia**	
	Firmicutes	**Staphylococcus**	
		**Streptococcus**	
		*Lactobacillus*	Improves skin moisture, color, texture, pores, wrinkles, UV spots, and brown spots [62] Antipathogenic function [63]
		**Finegoldia**	
		**Aerococcus**	
		**Anaerococcus**	
	Proteobacteria	Paracoccus	
		**Haematobcter**	
		**Sphingomonas**	
		**Hemophilus**	
	Bacteroidetes	**Flavobacterium**	
		**Prevotella**	

## Data Availability

Not applicable.
